# Is DBM Beneficial for the Enhancement of Bony Consolidation in Distraction Osteogenesis? A Randomized Controlled Trial

**DOI:** 10.1155/2015/281738

**Published:** 2015-01-29

**Authors:** Sang-Heon Song, Sang-Gyun Kim, Sung-Eun Kim, Hae-Ryong Song

**Affiliations:** ^1^Department of Orthopaedic Surgery, Myongji-Hospital, 55 Hwasu-ro, 14 Beon-gil, Deokyang-gu, Goyang-si, Gyeonggi-do 412-826, Republic of Korea; ^2^Department of Orthopaedic Surgery and Rare Diseases Institute, Korea University Medical Center, Guro Hospital, 80 Guro-Dong, Guro-gu, Seoul 152-703, Republic of Korea

## Abstract

The aim of the present study was to compare the radiographic and clinical outcomes of DBM injection and conventional treatment during tibial lengthening over an intramedullary nail in adult patients with short stature. Twenty-nine patients were randomized to receive DBM injection (*n* = 14) or conventional treatment without any injection (*n* = 15) and evaluated. The outcome was measured on the basis of the pixel value ratio (PVR) in the digital radiographs during the consolidation period; healing index; clinical assessment; and the rate of complications. In the DBM group, the mean PVR of 1 (mineral density of the callus is comparable to the adjacent bone) was reached by 40 weeks in anterior and medial cortices which was significantly different than that in the control group (*P* = 0.03 for anterior cortex; *P* = 0.04 for medial cortex). The average healing index in the DBM group was 39.8 ± 5.3 days/cm compared to 44.3 ± 5.8 days/cm in the control group (*P* = 0.05). There were no significant differences in clinical outcomes (*P* = 0.23) and functional status (*P* = 0.47) including complications (*P* = 0.72) between two groups. In this randomized clinical trial, injection of DBM at the time of initial operation enhanced consolidation of regenerate callus without interfering with clinical outcomes compared to that with conventional treatment.

## 1. Introduction

Demineralized bone matrix (DBM) is bone graft substitute that is washed, demineralized with organic solvents, dried, prepared, and sterilized. As first described by Urist et al. [1, 2], DBM may be generated by the acid extraction of processed allograft bone, giving rise to a demineralized matrix consisting of osteoconductive type 1 collagen and noncollagenous proteins, including osteoinductive bone morphogenetic proteins (BMPs) that stimulate the formation of bone at a defect site.

The first DBM/carrier products were introduced in 1991 and have since become one of the most widely used alternative graft products in the fields of spinal and dental surgery, and there are at least eight manufacturers with more than six types of carriers and 25 products in the market [3–5]. However, based on the literature review, there are only a few studies demonstrating the effect of DBM on the outcome of long bone applications [5–11]. And no clinical or radiographic comparative study has been reported, especially in the model of distraction osteogenesis.

In the current study we tested the hypothesis that an initial injection of DBM at the osteotomy site in tibial lengthening over an intramedullary nail would (1) enhance maturation of regenerate callus, (2) reduce the time to bony consolidation; (3) not influence functional outcomes and complications. The aim of the present randomized prospective study was to compare the radiographic and clinical outcomes of DBM injection and conventional treatment without any injection during tibial lengthening over an intramedullary nail in adult patients with short stature.

## 2. Materials and Methods

This was a single center, randomized, prospective, single blinded, parallel-group study conducted at the Department of Orthopaedic Surgery of the respective hospital. The study was approved by our institutional review board prior to initiation of the study, and informed consent was obtained from each patient prior to participation in the study. The study was registered as a clinical trial with the institutional review board of the respective hospital.

From January 2010 to January 2011, 39 patients underwent bilateral tibial lengthening at our institution and were evaluated for eligibility for participation in the study. Of these 39 patients, 6 patients did not match the inclusion criteria. Three patients refused randomization, and insisted on receiving conventional treatment, and hence they were excluded from the study. The remaining 30 patients met the inclusion criteria, agreed to participate in the study, and were enrolled in the study. Fifteen patients were randomized to the DBM group, and another fifteen patients were randomized to the conventional group (control group). One patient in the DBM group was lost to follow-up and hence was excluded from the study. The remaining 29 patients (22 men and 7 women) with a mean age of 22.8 years (range, 18 to 33 years) completed the study. A tibia among two limbs of each patient was randomly selected and evaluated to minimize bias [12]. Thus, 14 patients in the DBM group and 15 patients in the control group were available for follow-up.

### 2.1. Inclusion and Exclusion Criteria

The indication for surgery was idiopathic or familial short stature with height less than five percentile for age and gender. The inclusion criteria were (1) age between 18 and 40 years, (2) those who underwent bilateral tibial lengthening over an intramedullary nail, and (3) the amount of tibial lengthening between 60 mm and 80 mm or 20~25% of the initial tibial length. The patients who had (1) serum phosphate and calcium levels less or more than 30% of normal range, (2) received NSAIDs, systemic steroids or antithrombotic agents, (3) had a history of previous fracture, infection or tumor, and (4) had an addiction to alcohol or heavy smoking (more than 2 packs per day) were excluded from the study.

### 2.2. Sample Size Calculation

Before the study, a sample size analysis was performed. The sample size was chosen on the basis of the healing index after a thorough consultation with a professional biostatistician. With the alpha level set at 0.05, it was determined prospectively that 12 participants per group would give 90% power to identify a difference in healing indices with an unpaired *t*-test. So we decided to enroll 15 patients per each group as considering the possibility of follow-up loss or some inevitable personal conditions that make it excluded during the study.

### 2.3. Randomization and Blinding

The clinical research nurse coordinator explained the workflow of the study. After the study nurse had obtained patients' consent, the patients were randomized to either the DBM group or the control group using sequentially numbered, sealed envelopes prepared by a resident who was not involved in the study. While surgical teams were aware of allocation of the patients; patients, outcome assessors, and data analysts were kept blinded to the allocation.

### 2.4. Operative Treatment

**Figure 1:**
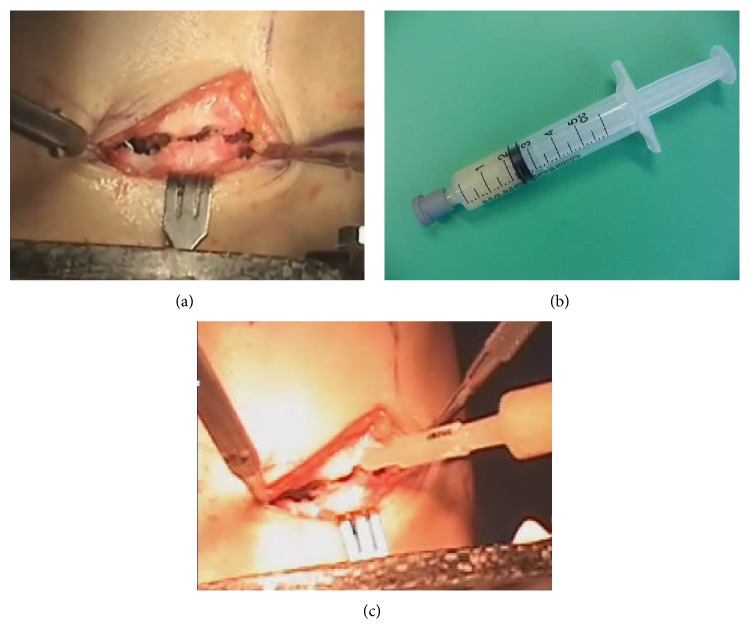
Clinical photo shows how to apply injectable DBM to the osteotomy site at the initial operation. (a) After proximal tibial osteotomy using multiple drill hole technique, (b) putty type DBM in a 5 cc sized syringe, and (c) DBM was injected at the osteotomy site.

All patients were operated on by the senior author (HRS). Our technique was similar to that described by previous authors [13–15] and AO tibial interlocking nails and Ilizarov external fixators were used. Three rings were used for distraction. Two proximal wires and one proximal half-pin were inserted and fixed at the proximal ring. Two distal wires and one distal half-pin were inserted and fixed at the distal ring. The middle ring had no half-pin and wire. For the patients allocated to the DBM group, 3 cc of DBM putty (ExFuse, Hanmi Pharmaceutical Co., Ltd., Seoul, Korea) was injected at the osteotomy site at the time of initial operation (Figure 1). Patients underwent supervised daily physiotherapy including active and passive range of motion exercises for the knee and ankle starting at 2 days after surgery. Physiotherapy was performed twice per day for 2 hours during 2 weeks of admission and for 1 hour per day after discharge until the end of the distraction phase. Daily walking at least 4 hours with partial weight bearing with two crutches was allowed during the distraction phase. Distraction was started 7 days postoperatively at a rate of 0.25 mm four times a day until the desired length was achieved. After the desired limb length was achieved, distal locking screws were inserted and the external fixator was removed and partial weight bearing was allowed when the pixel value ratio [14, 16] was 1 in two cortices among four cortices. Full weight bearing without crutches was allowed when the pixel value ratio was 1 in three cortices, based on the previous study [14].

### 2.5. Outcomes

All patients were assessed radiographically and clinically every month after the initial operation.

**Figure 2:**
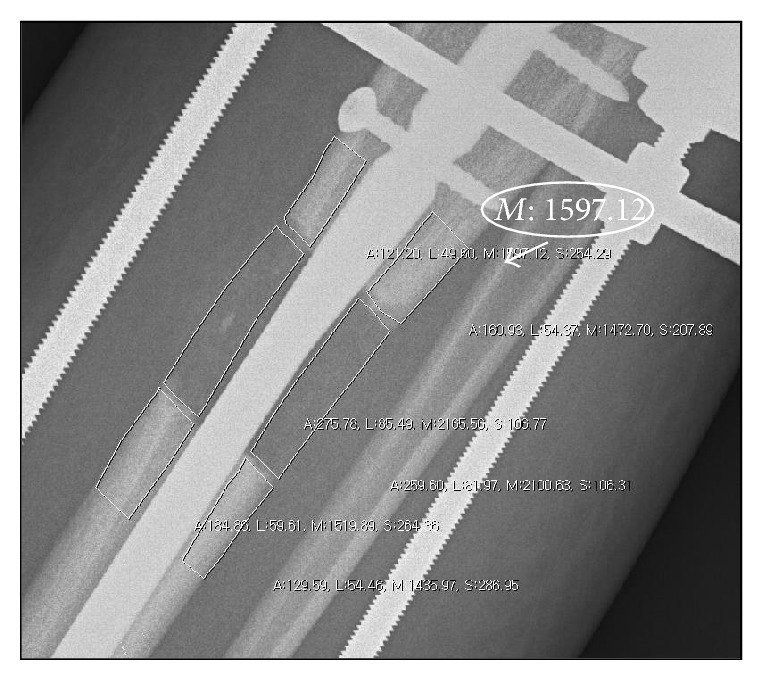
The radiograph shows how to measure the pixel values on a digital radiograph. The different cortical segments of callus and the proximal and distal segments were measured with use of the free region of interest methods of StarPACS PiView Star 5.0.6.1 software (Infinitt Co., Ltd., Seoul, Korea). White circled “*M*” value was the pixel value of the region of interest drawn.

Primary outcome measure was the pixel value ratio (PVR) [14, 16–18] with respect to the callus maturation until full consolidation in the digital radiograph of tibial AP and lateral views. The pixel value ratio was measured on digital radiogram with StarPACS PiView Star 5.0.6.1 software (Infinitt Co., Ltd., Seoul, Korea) in order to quantify callus maturation at every month during the consolidation period from the end of distraction until the end of this study. The pixel value was measured using the pixel lens included in the tools of the program. The pixel values of the proximal, distal, and regeneration areas were calculated from the mean value of each area using the free region of interest method (Figure 2). Care was taken to avoid any metal wire (hardware shadow). The areas of the regenerate proximal and distal bony fragments were divided into anterior, posterior, medial, and lateral areas, which were between the outer margins of the nail and cortices. The PVR of the regenerate then was calculated using the following formula: pixel value ratio = [(pixel value of the proximal segment + pixel value of the distal segment)/2]/pixel value of the regenerate. Since we measured the raw pixel value, which is inversely related to radiodensity (i.e., as radiopacity increases the pixel value decreases), an inverse ratio is used in the above formula. The stage of corticalization was decided by PVR. A PVR of 1 indicated that corticalization of the regenerate in the lengthening area was comparable to that of the adjacent bone. Less than 1 meant lesser maturation of the regenerate.

Two assessors (one orthopaedic surgeon and one radiologist), who were not involved in the surgical treatment and were blinded to the patients' allocation measured PVR twice per each radiograph and the mean of the four values was used for the analysis.

For secondary outcome measure, healing index of each group was evaluated and functional assessment using Short Form 36 (SF36) questionnaire was done at the time of the initial visit and the final visit. Full consolidation for the evaluation of healing index was defined as an extracortical bridging callus on three of four cortices as viewed on anteroposterior and lateral radiographs. Complications were monitored and classified into minor and major according to Paley classification [19]. The major complications interfered with the original goals of treatment and the minor complications did not interfere with the original goals of treatment.

### 2.6. Statistical Methods

For the primary outcome measure of PVR, changes in PVR per month were analyzed using the Wilcoxon rank sum test to compare between the two groups at each time point. For the values of the secondary outcome measures (healing index, SF36 scores, and complication rate), an unpaired *t*-test was used for the determination of differences in the mean values between the two groups. All statistical tests were performed using the SPSS (SPSS for Windows Release 12.0; SPSS Inc., Chicago, IL, USA), and *P* values of <0.05 were considered significant. Additional post hoc power analysis was done for the statistically significant values between two groups using G∗Power (3.1.9.2 for Windows; Faul, Erdfelder, Lang & Buchner, Kiel, Germany) [20, 21] to test the design of this study.

## 3. Results

### 3.1. Demographic Characteristics

**Figure 3:**
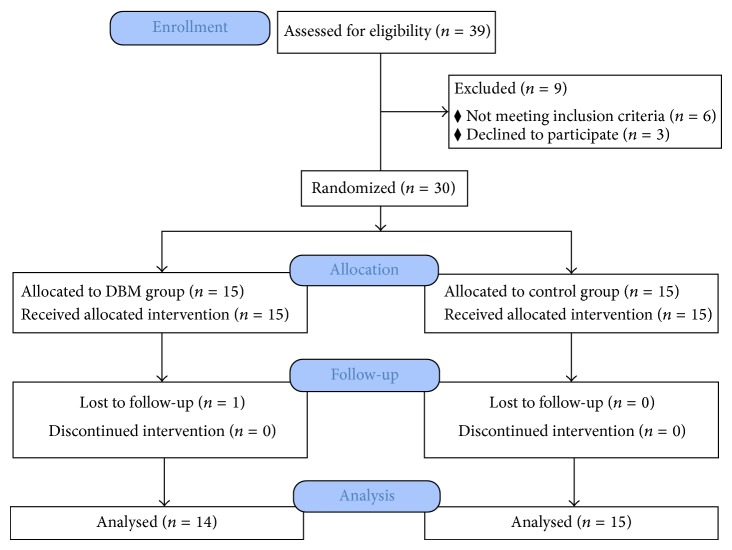
Flowchart showing the CONSORT (Consolidated Standards of Reporting Trials) diagram of the flow of participants through the study.

From January 2010 to January 2011, thirty patients who underwent bilateral tibial lengthening over an intramedullary nail for short stature at our department were randomly enrolled in the study. One patient in the DBM group was lost to follow-up, leaving 29 patients for the analysis (14 patients in the DBM group and 15 patients in the control group) (Figure 3). The mean age of the patients in the DBM group and the control group was 23.1 ± 4.1 years and 21.6 ± 3.4 years. The mean weight of the patients in the DBM group and the control group was 76.7 ± 12.3 kg and 78.1 ± 11.7 kg, respectively. The amount of lengthening (percentage) in the DBM group and the control group was 6.8 ± 1.0 cm (23.8 ± 2.5%) and 7.0 ± 1.0 cm (24.0 ± 2.4%), respectively (Table 1).

### 3.2. Radiographic Results

**Figure 4:**
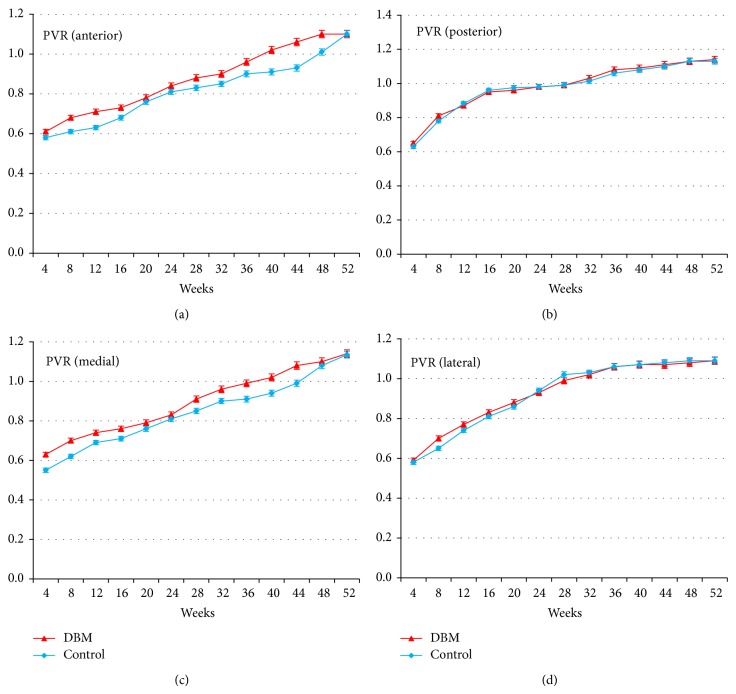
(a)–(d) Each graph shows the mean pixel value ratio (PVR) in four different cortical segments for two groups. At the visit of 40 weeks, the mean PVR of anterior (a) and medial (c) cortices reached 1 in DBM group; however, the mean PVR of anterior (a) cortices reached 1 in 48 weeks and 44 weeks for medial (c) cortices in control group. The mean PVR of anterior (a) cortices at 40 weeks was 1.02 (95% confidence interval, 0.94 to 1.11) for the DBM group and 0.91 (95% confidence interval, 0.86 to 0.98) for the control group. And the mean PVR of medial (c) cortices at 40 weeks was 1.01 (95% confidence interval, 0.93 to 1.09) for the DBM group and 0.93 (95% confidence interval, 0.82 to 1.01) for the control group. There were no significant differences for the mean PVR of posterior (b) and lateral (d) cortices between two groups.

The mean PVR increased progressively during the period of consolidation, more so in lateral and posterior cortices and less so in anterior and medial cortices in the two groups. In the control group, the mean PVR of 1 (mineral density of the callus is the same as that of the bone in the proximal and distal segments) was reached by 28 weeks in lateral cortices, by 32 weeks in posterior cortices, by 44 weeks in medial cortices, and after 48 weeks in anterior cortices. In the DBM group, the mean PVR of 1 was reached by 40 weeks in anterior and medial cortices which was significantly different than that in the control group (*P* = 0.03 for anterior cortex; *P* = 0.04 for medial cortex; Wilcoxon rank sum test). The mean PVR of the anterior cortices at 40 weeks was 1.02 (95% confidence interval, 0.94 to 1.11) in the DBM group, and 0.91 (95% confidence interval, 0.86 to 0.98) in the control group (94.2% post hoc power). And the mean PVR of the medial cortices at 40 weeks was 1.01 (95% confidence interval, 0.93 to 1.09) in the DBM group and 0.93 (95% confidence interval, 0.82 to 1.01) in the control group (74.5% post hoc power). But the mean PVR of 1 was reached by 28 weeks in lateral cortices and by 32 weeks in posterior cortices which was not significantly different compared to that in the control group (*P* = 0.47 for lateral cortex; *P* = 0.51 for posterior cortex; Wilcoxon rank sum test) (Figure 4).

The mean healing indices of the DBM group and the control group were 39.8 ± 5.3 days/cm (95% confidence interval, 33.9 to 44.2 days/cm) and 44.3 ± 5.8 days/cm (95% confidence interval, 37.7 to 50.1 days/cm), respectively, and this difference was statistically significant (*P* = 0.05; unpaired *t*-test) (55.6% post hoc power) (Table 2).

### 3.3. Clinical Results and Complications

The mean SF36 scores were similar between the two groups and the differences between baseline scores and postoperative scores in each component summary were similar between the two groups (Table 2). The complication rate per segment in the DBM group and the control group was 78% (11/14) and 80% (12/15), respectively, without any statistically significant difference (Table 2). There were 11 complications, of which 10 were minor complications and 1 was a major complication in the DBM group. No deep intramedullary infections or other systemic adverse effects were reported. The minor complications were 8 pin tract infections of grade 1 and 2. All pin tract infections responded well to local pin site care and oral antibiotics. Wire breakage occurred in one segment during the consolidation stage, and this wire was removed in the outpatient clinic and there was no need for inserting another wire as the regenerate was well consolidated. Another minor complication was premature fibular consolidation and valgus angulation which was treated with osteoclasis of the fibular osteotomy site and proximal transfer of the fibula with additional fibular half pin insertion. The major complication was compartment syndrome which occurred immediately postoperatively and was managed by fasciotomy without any residual effect. Ankle equinus contracture was observed in two patients and was treated with gastrocnemius-soleus intramuscular aponeurotic recession without any effect on the muscle activity. There were also 12 minor complications in the control group, of which 10 were pin tract infection of grade 1 or 2 and the other two were fibula-related complications (1 nonunion, 1 premature consolidation).

## 4. Discussion

DBM is mainly comprised of collagen (93%), which provides an osteoconductive surface. Soluble proteins, such as osteoinductive BMPs and a growth cocktail of synergistic proteins (transforming growth factor beta, insulin-like growth factor, platelet-derived growth factor, and fibroblast growth factor), only represent approximately 5% of DBM. The remaining 2% of DBM is made up of residual mineralized matrix. In addition to its osteoinductive ability, DBM also supports new bone formation via osteoconductive mechanisms [4, 22–24]. In most available literatures, the effect of DBM as a bone graft substitute compared to that of iliac crest bone graft was demonstrated in spinal fusion surgery. And the results were variable from a superior or comparable to those of an autograft to a negative effect with a higher rate of pseudoarthrosis [25–30]. However, only a few clinical studies have demonstrated the effect of DBM on the outcome of long bone applications, mostly in the cases of nonunion [5–10]. The present study demonstrated that the initial injection of DBM at the osteotomy site enhanced healing of the regenerated callus as compared to that with the conventional treatment in tibial lengthening over an intramedullary nail. To the best of our knowledge, the present study is the only and the first randomized, controlled, clinical trial that has attempted to assess the effect of DBM in the long bone distraction osteogenesis model.

The present study certainly has some limitations. Firstly, it is confined to the young adults with a single disease who may have a good bone stock and adequate soft tissue envelope which may lead to sufficient new bone formation without any further cost-ineffective interventions. Secondly, the follow-up period of this study was 18 months. Even though this follow-up period was sufficient to test the hypothesis proposed in this study design, a longer term follow-up study is needed to assess the possible systemic complications related to the use of DBM or the mechanical strength of regenerate bone. Thirdly, the radiodensity of DBM itself might influence the results of pixel values obtained from the radiographs. Clinically, DBM shadows were identifiable in the initial radiograph, but the shadows were relatively diluted at the end of distraction periods. In this study, the radiodensity of DBM could be hardly differentiated after the time of around 20 to 24 weeks. According to the findings of PVRs in each cortex (Figure 4), PVRs of DBM group were initially higher than those of control group in all cortices, but showed similar values at the time of 20 to 24 weeks. After that, PVRs of DBM group showed faster inclinations than those of control group in anterior and medial cortices. We assumed that even though the radiodensity of DBM was seen in the initial periods, there was no further adverse effect on the reading of PVR in the radiograph due to the DBM shadows in the midphase and the late stage of callus maturations in this study. Lastly, using this study protocol, it is not possible to find out whether DBM truly has osteoinductive, osteogenic potential, or osteoconductive capability for new bone formation. However, the clinical significance of this study is that this study protocol successfully demonstrated about 11% increase in the healing index with massive lengthening of 6 to 8 cm during distraction osteogenesis which could reduce the time-consuming period of external fixation. And the primary outcome measure of the PVR showed a significant increase in the anterior and medial cortical segments, in which delayed callus formation can be commonly observed than in the lateral or posterior cortical segments due to a relatively poor soft tissue envelope even after meticulous repair and preservation of the periosteum after corticotomy at the time of surgical procedure. These findings may have important clinical relevance for the treatment of short stature when a large amount of lengthening is indicated.

The aim of this study was to compare the effect of DBM and conventional treatment in tibial lengthening over an intramedullary nail in terms of radiographic outcome and functional assessment and complications. The primary outcome measure of PVR showed a significantly enhanced callus maturation in the anterior and medial cortices in the DBM group and comparable maturation in the lateral and posterior cortices in the control group. And the secondary outcome measures of healing index demonstrated about 11% increase in consolidation of regenerate callus in the DBM group without interfering with the SF36 functional outcomes or complications than that in the control group. Wilkins and Kelly reported that percutaneous use of a mixture of autologous bone marrow and allograft DBM led in 61 to 69 patients with stiff nonunions of long bones to union in an average period of 8.1 months [8]. Also Hierholzer et al. reported that clinical and radiological union was achieved in 97% in the DBM (Grafton) group in 4.2 months for the treatment of diaphyseal nonunion of the humerus [6]. Although there was no available literature dealing with distraction osteogenesis for long bone applications, the results of our study are comparable with those of the previous studies and they may set the standard for further studies with a high level of evidence.

Among the more than 25 commercially available DBM products, there are various forms of preparations, washing procedures, sterilization methods, and storage conditions with different types of carriers (glycerol, poloxamer, gelatin, calcium sulfate, lecithin, hyaluronic acid, and carboxymethyl cellulose) and DBM products are available in a number of different forms (powder, putty, chips, crushed granules, or gel-filled syringes) [5, 31]. Based on the literature review, there were at least three studies [5, 9, 32] that compared the clinical efficacy of different DBM products, mainly between Grafton (DBM in a glycerol carrier) and Orthoblast (DBM with a reverse thermal poloxamer carrier). The reported success rates for these products varied from 52% to 100%. The DBM product used in our study comprised of a mixture of DBM and a carboxy methyl cellulose (CMC) carrier and was a putty injectable type of DBM. CMC has been utilized as a pharmacological additive in many drugs. It is biodegradable and nontoxic and does not have any untoward effect on bone healing. The fact that CMC has a semisolid consistency and solidifies later makes its handling easier and prevents spillage while applying it to the defect site. Even though there are some benefits of an injectable DBM product such as easy handling at the time of surgical intervention, the results of this study did not show any superiority of the DBM-CMC product over the other DBM products and thus further studies evaluating the most appropriate and effective composite material carrier for enhancement of new bone formation in the orthopaedic field are needed.

## 5. Conclusions

In conclusion, this study demonstrated that the primary outcome measure of PVR showed a significantly enhanced callus maturation in the anterior and medial cortices in the DBM group and comparable maturation in the lateral and posterior cortices in the control group. And the secondary outcome measure of healing index demonstrated about 11% increase in consolidation of regenerate callus in the DBM group. So, DBM is clinically beneficial when a large amount of tibial lengthening is indicated in patients with idiopathic short stature or familial short stature.

## Figures and Tables

**Table 1:** Patient characteristics^*^.

	DBM group (*N* = 14)	Control group (*N* = 15)
Sex (number of patients)		
Male	11	12
Female	3	3
Age^#^ (years)	23.1 ± 4.1	21.6 ± 3.4
Weight^#^ (kg)	76.7 ± 12.3	78.1 ± 11.7
Tibial length gain (cm)	6.8 ± 1.0	7.0 ± 1.0
Tibial length gain (%)	23.8 ± 2.5	24.0 ± 2.4

(^*^The differences between the groups were not significant for all parameters. ^#^The values are given as the mean and the standard deviation.).

**Table 2:** Clinical results including mean healing index, complication rate per segment, and the short form 36 questionnaire (SF-36).

	DBM (*N* = 14)	Control (*N* = 15)	*P* value^*^
Mean healing index (days/cm)	39.8 ± 5.3	44.3 ± 5.8	0.05
SF-36 (PCS) gain after operation	10.3 ± 2.6	11.9 ± 2.3	0.23
SF-36 (MCS) gain after operation	14.8 ± 4.2	15.9 ± 4.8	0.48
SF-36 (TCS) gain after operation	19.3 ± 6.1	18.9 ± 5.9	0.47
Complication rate per segment (%)	78	80	0.72

(^*^Unpaired *t*-test; PCS, physical component summary; MCS, mental component summary; TCS, total component summary).
